# Legislation for trial registration and data transparency

**DOI:** 10.1186/1745-6215-11-64

**Published:** 2010-05-26

**Authors:** Zhao-Xiang Bian, Tai-Xiang Wu

**Affiliations:** 1 School of Chinese Medicine, Hong Kong Baptist University, Hong Kong, China; 2 Department of Clinical Epidemiology, Chinese Evidence-based Medicine Center, West China Hospital, Sichuan University, Chengdu 610041, Sichuan Province, China

## Abstract

Public confidence in clinical trials has been eroded by data suppression, misrepresentation and manipulation. Although various attempts have been made to achieve universal trial registration- e.g., Declaration of Helsinki, WHO clinical Trial Registry Platform (WHO ICTRP), the International Committee of Medical Journal Editors requirement- they have not succeeded, probably because they lack the enough power of enforcement.

Legislation appears to be the most efficient and effective means to ensure that all researchers register their trials and disseminate their data accurately and in a timely manner. We propose that a global network be established. This could be accomplished in two steps. The first step is to legislate about trial registration and data transparency, such as USA's FDAAA Act 2007; and the second step to establish a global network to ensure uniform, international consistency in policy and enforcement of trial registration and data transparency.

## Introduction

Public confidence in clinical trials has been eroded by high-profile scandals involving alleged data suppression, misrepresentation and manipulation [1-6]. Such misconduct has serious implications for the health care system, and also violates the ethical responsibilities of researchers and sponsors [7,8]. These scandals have highlighted the need and generated the impetus to increase data transparency in order to restore public confidence in clinical trials [9,10]. Unfortunately, the trial registration cannot adequately ensure full data transparency.

### Trial registration

The concept of trial registration was first mentioned by Simes RJ in 1986[11]. At that time, the major purpose was to reduce publication bias [12]. The first registry http://ClinicalTrials.gov was launched under Section 113 of the Food and Drug Administration Modernization Act (FDAMA 113) of the United States (US) in 1997 [13]. Unfortunately, despite legislation, many drug trials in the US were not registered [14]. The inadequate reporting in the US gained attention in 2004 when scandals arose over selective reporting of trial data for selective serotonin reuptake inhibitors (SSRIs) [15] and rofecoxib [16]. The SSRIs case resulted in the New York State attorney suing GlaxoSmithKline (GSK) company over its 'illegal and deceptive' reporting by suppressing reports of suicidal thinking among patients and misleading doctors into overprescribing the antidepressant paroxetine to children [17]. Eventually, the New York State Attorney General's office reached an agreement with GSK in which GSK was required to maintain an online clinical trials registry that included information about safety and efficacy, type and severity of side effects, midstream methodological changes and early terminations [18]. Unfortunately, this settlement did not change the registration requirements for trials of other US drug companies or for trials that were not supported by drug companies. Of course, it had little effect on trials conducted in other countries.

The first international policy on trial registration was introduced by the International Committee of Medical Journal Editors (ICMJE) in October 2004[19]. The policy required that trials be registered at the http://ClinicalTrials.gov site before enrolment of patients in order for the manuscript to be considered for publication in ICMJE journals. This action had 'mandatory' enforcement power, and the number of trial registrations at http://www.ClinicalTrials.gov increased dramatically after the ICMJE announcement [20]. In 2007 ICMJE updated their statement to call for prospective registration in the ICMJE-endorsed registry for publication in any of their member journals [21]. However, publication in ICMJE journals is sometimes not an objective for conducting a clinical trial, therefore the policy has limited power to secure universal trial registration.

In the Ministerial Summit on Health Research that took place in Mexico City, Mexico, in November 2004, the World Health Organization (WHO) called for members to *"establish a voluntary platform to link clinical trials registers in order to ensure a single point of access and the unambiguous identification of trials with a view to enhancing access to information by patients, families, patient groups and others"*. Currently, the WHO ICTRP, established in August 2005, is composed of Primary Registries (currently 10) and Partner Registries; and it provides an international platform that enables researchers, health practitioners, consumers, journal editors and reporters to search more easily and quickly for information on clinical trials [22]. But WHO ICTRP is still a voluntary system, relying on the consciences of researchers for compliance, as it says, 'The registration of all interventional trails is a scientific, ethical and moral responsibility.'

The Ottawa Statement, which was published in 2005, required that registration and early public release of accurate information about all trials are necessary to fulfill ethical obligations to participants; and all trial results should be registered and made publicly available, along with sufficient protocol information to enable critical assessment of their validity[23]. However, the Statement only recognizes that trial registration is an ethical obligation. Unfortunately, previous scandals clearly demonstrate that ethical power alone is not enough to persuade all trial researchers to register their trials.

In 2008, the World Medical Association announced in the revised Declaration of Helsinki that "*Every clinical trial must be registered in a publicly accessible database before recruitment of the first subject" *[24]. In this way the World Medical Association has unequivocally established its position that public registration of clinical trials is necessary. If the ethics committees, at every research institution in every country, would strictly follow the Declaration, it would represent a necessary but not sufficient condition toward securing universal trial registration. One big problem is that not all trials pass through a strict ethics committee approval process [25], and also it is pending question whether Declaration of Helsinki could be completely followed by the ethics committee.

In summary, the current requirement about trial registration, majorly from ethical aspects and publication purpose, does not have the universal power to make sure all trials registered.

### Data transparency

The purpose of trial registration is to make sure all data about a trial is available generally to the public and specifically to health care professionals. It is an original motivation behind the trial registration, especially the 2004 call for a central registry. But trial registration itself does not automatically ensure data transparency. Even if a trial registration requirement is implemented successfully in each country in the world, it still cannot secure data transparency. The reason is that registration requires only minimal information; generally it simply establishes there is a trial currently underway or about to start somewhere in this world. After registration, trial investigators should update their entries, especially releasing the trial results in an accurate and timely manner. But recently studies showed that trial registration does not ensure the timely availability of accurate trial results [26]. In the study, 57 endometriosis-related clinical trials registered at ClinicalTrials.gov were found, with 25 listed as completed, and 2 as suspended; leaving 30 unaccounted. Among 57 trials, there are 15 completed phase II/III trials, yet only three of the 15 trials (20%) had published their results. The remaining 12 (80%) studies so far have not published their findings even though some of these trials were completed as long as seven years. Clearly, trial registration requirements do not result in data transparency automatically. A full requirement for trail registration should let the trialists i) to register a trial before starting the patient recruitment, ii) to update the modification of trial protocol if any, and iii) to disseminate the trial results accurately and in a time manner. Therefore, trial registration should have a package of requirements from the start to the end, thus to make the public have possibility to know the whole profile of a trial.

### Legislation

Recent past years have witnessed the ineffectiveness of voluntary, partly-mandatory, and ethical requirements for trial registration. Such requirements cannot ensure that all trials are registered and that data are transparently reported. The major reason is that these requirements are lack of enough enforcement for trialists. Only a mandatory system can effectively ensure global trial registration and data transparency. The best mandatory system is legislation. Only legislation has universal power to ensure that all trial researchers register their trials and disseminate their data timely and concisely. The agreement between the New York State Attorney General' office and GSK company about trial is a good example.

After the SSRIs scandals, the US Congress enacted the Food and Drug Administration (FDA) Amendments Act of 2007 (FDAAA) in 2007, which mandates public registration and disclosure of results of 'applicable' clinical trials of drugs, biologics and devices on http://www.Clinicaltrials.gov[27]. Starting from December 2007, results disclosure occurred in three stages initially with links to information from the FDA and NIH about FDA-approved products as well as Medline citations. A Basic Results Database appeared in September 2008, and an Expanded Results Database will appear in 2010. The Act FDAAA 2007 requires that all results for trials of FDA-approved products must be posted within 12 months of trial completion. These requirements targeted "applicable" trials of drugs and devices subject to FDA regulation, but excluded Phase I drug trials and device feasibility studies. This new law requires researchers not only register trials but also put results into the database on time. Clearly, the Act has the power to require researchers in the US to register their trials. But limitations exist in that not all FDA-approved drug trials need to be registered. Also, this requirement may have an effect on publication bias [28]. Some nations such as India, not just USA, have made great efforts to legislate, thus to make sure all trials be registered [29]. We hope to see more and more nations take necessary actions for this purpose.

### Global network

Legislation for the trial registration in a few countries is not enough and a global network for the trial registration should be established. First, each country that has the ability to conduct clinical trials should have such legislation. It is conceivable that if one country requires trial registration and data transparency, while another country does not, then there is a possibility that many, if not most, clinical trials may shift to that country. Second, in order to have a universal mandatory system for trial registration and data transparency, based on the requirements from WHO ICTRP, ICMJE, and the Declaration of Helsinki, a network involving all countries should be established to ensure uniform, international consistency in policy and enforcement of trial registration and data transparency.

This network could be established in regional basis, and a global network could be based on these regional networks. It should be emphasized that local effort from one country to a region is the basis for the global network. In this process, WHO may form an international advisory committee to provide consultation for each country and to help them to meet this aim. Further, this advisory committee can be charged with the responsibility of monitoring the implementation of such legislation through different approaches, such as publishing white paper of trial registration annually, etc.

When this global network is available, all trials could be traced and data could be searched, thus ensuring that the data are fully transparent to the public.

## Conclusion

Existing efforts for trial registration and data transparency are not enough. They cannot compel all trial investigators to register their trials, and disseminate their trial results in an accurate and timely manner. Each country should legislate to develop a mandatory system, and a network involving all countries should be established, thus ensuring data transparency to restore the public confidence in the clinical trial. This system would best serve the interests of the public, the research community, and patients who are, ultimately, ourselves.

## Competing interests

The authors declare that they have no competing interests.

## Authors' contributions

ZXB initiated and prepared the manuscript. TXW revised and added some valuable points into it. All authors read and approved the final manuscript.
